# 1641. Prescribing and Dispensing Delayed Antibiotics to Pediatric Patients with Acute Otitis Media and Pharyngitis

**DOI:** 10.1093/ofid/ofad500.1475

**Published:** 2023-11-27

**Authors:** Shobhitha Balasubramaniam, Melanie Melanie, Eleanor Fitzpatrick, Katrina Hurley, Emily Black

**Affiliations:** Dalhousie University, Halifax, Nova Scotia, Canada; IWK Health, Halifax, Nova Scotia, Canada; IWK Health, Halifax, Nova Scotia, Canada; IWK Health, Halifax, Nova Scotia, Canada; Dalhousie University, Halifax, Nova Scotia, Canada

## Abstract

**Background:**

Delayed antibiotic prescriptions with instructions to wait a defined period of time before filling the prescription is a strategy often used to decrease the unnecessary use of an antibiotic. Although "watchful waiting" for illnesses, such as acute otitis media (AOM) and pharyngitis, that are suspected of being viral is recommended, little is known about how often delayed antibiotics are prescribed at our institution. The primary objectives of this study were to characterize delayed prescriptions for pediatric patients with AOM or pharyngitis and to assess each prescription for adherence to guidelines.

**Methods:**

Design: This study was a retrospective chart review. Pediatric patients with AOM and pharyngitis assessed and discharged from the Emergency Department (ED) between March 1, 2019 to February 28, 2021 were considered for inclusion. Data were collected from the ED Treatment Record by a trained research assistant.

Setting: Our study was completed in a pediatric ED at a tertiary hospital in Canada.

Exposures or interventions: Patients prescribed a delayed antibiotic on discharge from the ED.

Main Outcome Measures: Frequency of delayed antibiotic prescribing, proportion of antibiotic prescriptions adherent to guidelines for empiric choice, dose, and duration. Adherence to guidelines was assessed using local guidelines and position statements by the Canadian Pediatric Society as the gold standards.

Statistical analysis: Data were entered in REDCap and exported to excel for descriptive analysis.

**Results:**

Delayed antibiotics were prescribed to 21.2% (332/1563) of patient encounters during the study period. Amoxicillin was the most commonly prescribed antibiotic (88.6%, 294/332). The majority of delayed prescriptions were prescribed for AOM (77.7%, 258/332). Most patients (94.8%, 309/326) received a delayed prescription for an empiric antibiotic that adhered to guidelines. Fewer prescriptions adhered to guidelines for dose (75.8 %, 247/326) and duration (73.6%, 240/326).

**Conclusion:**

Most patients received the appropriate empiric antibiotic to treat AOM and pharyngitis however, dose and duration of use may be improved through antimicrobial stewardship initiatives.

**Disclosures:**

**Emily Black, BSc(Pharm), PharmD**, Drug Evaluation Alliance of Nova Scotia: Grant/Research Support|Research Nova Scotia: Grant/Research Support

